# A Functionalized Tetrakis(4-Nitrophenyl)Porphyrin Film Optical Waveguide Sensor for Detection of H_2_S and Ethanediamine Gases

**DOI:** 10.3390/s17122717

**Published:** 2017-11-24

**Authors:** Gulimire Tuerdi, Nuerguli Kari, Yin Yan, Patima Nizamidin, Abliz Yimit

**Affiliations:** College of Chemistry and Chemical Engineering, Xinjiang University, Urumqi 830046, China; gulmirat@126.com (G.T.); nurri7695@163.com (N.K.); yanyinwx@163.com (Y.Y.); patima207@aliyun.com (P.N.)

**Keywords:** nafion, tetrakis(4-nitrophenyl)porphyrin, ethanediamine, hydrogen sulfide, optical waveguide

## Abstract

The detection of hydrogen sulfide (H_2_S) and ethanediamine, toxic gases that are emitted from industrial processes, is important for health and safety. An optical sensor, based on the absorption spectrum of tetrakis(4-nitrophenyl)porphyrin (TNPP) immobilized in a Nafion membrane (Nf) and deposited onto an optical waveguide glass slide, has been developed for the detection of these gases. Responses to analytes were compared for sensors modified with TNPP and Nf-TNPP composites. Among them, Nf-TNPP exhibited significant responses to H_2_S and ethanediamine. The analytical performance characteristics of the Nf-TNPP-modified sensor were investigated and the response mechanism is discussed in detail. The sensor exhibited excellent reproducibilities, reversibilities, and selectivities, with detection limits for H_2_S and ethanediamine of 1 and 10 ppb, respectively, and it is a promising candidate for use in industrial sensing applications.

## 1. Introduction

Ethanediamine (EDA) is a common volatile organic compound (VOC) [1], and the National Institute for Occupational Safety and Health recommends an occupational exposure limit of under 10 ppm [2]. Potential symptoms of overexposure to EDA include nasal and respiratory system irritation, asthma, as well as liver and kidney damage. EDA is very soluble in water, ethanol, and methanol, and it is employed as a reaction intermediate for the industrial production of pharmaceuticals, polymers, and dyes [3,4,5,6]. Recently, several studies directed toward the development of EDA gas sensors have been reported; for example, electrochemical [7,8] and luminescence sensors [9] exhibit high sensitivities toward EDA. However, they have drawbacks, such as poor detection limits, lack of mobility, and large gas volumes that are required for detection, which limits their use in environmental-monitoring departments. Castillero and co-workers produced a protonated porphyrin/TiO_2_ composite thin film for the determination of amines (ammonia, EDA, and butylamine, etc.) by monitoring spectral changes resulting from protonation-deprotonation reactions of porphyrins before and after exposure to amines and HCl [10]. While this sensor exhibited better sensitivity and response-recovery to analytes, its response and recovery times were long. Therefore, lower detection limits and faster response-recovery times for the detection of EDA is a meaningful objective.

On the other hand, hydrogen sulfide (H_2_S), which has an unpleasant smell that similar to that of rotten eggs, is toxic and corrosive [11]. Natural emissions of H_2_S into the atmosphere are predominantly related to the degradation of organic matter through the anaerobic reduction by bacteria [12]. Anthropogenic sources, such as fossil-fuel burning and petroleum extraction, result in substantial emissions of H_2_S into the environment [13]. Common H_2_S gas sensors are based on metal oxide semiconductors and various hybrid materials. For example, sensors based on porous CuO nanosheets exhibit detection limits of 0.7 to 10 ppb at room temperature, with response and recovery times for the detection of 200 ppb H_2_S of 336 s and 543 s, respectively [14]. Liu and co-workers prepared CuO-ZnO nanorods using a pulsed-laser-deposition method; these nanorods exhibited a 0.5 ppm detection limit for H_2_S gas at room temperature [15]. A H_2_S gas sensor, based on p-type CuO (111) nanocuboids, also exhibits a 1 ppm detection limit for H_2_S at 200 °C [16]. However, some drawbacks exist, including high operating temperatures, and the low sensitivities and selectivities of CuO, ZnO, Fe_2_O_3_, SnO_2_, and In_2_O_3_/ZnO, which limits the wide application of these types of novel nanomaterials. Highly sensitive low-cost sensing materials that can rapidly identify analytes are in high demand for multiple applications, such as environmental monitoring, medical diagnostics, and food-quality control. Porphyrins and related macrocycles have been intensively exploited as materials for use in chemical sensors [17], since these devices can exploit most of their physicochemical functions [18], such as reversible binding [19], catalytic activation [20], and optical changes [21]. The optical properties of porphyrinoids have attracted significant attention because they are applicable to the development of gas chemosensors, in which detection is based on the differential absorption of left- and right-hand circularly polarized light in the ultraviolet, visible, or IR region. Toxic gases, including VOCs [22,23], NO_2_ [24], CO_2_ [25], H_2_S [26], ammonia, and amines [27,28] modify the spectrum of the porphyrin, which is useful for optical gas sensing. However, intermolecular π-π interactions between porphyrin rings usually lead to aggregation that, in general, is detrimental for gas-sensing purposes. Porphyrins incorporated into Nafion (Nf), PVC (polyvinyl chloride), or nitrocellulose films have been found to be more sensitive to ammonia than other materials [29]. Among these, Nf has been used as a functional material due to notable features that include its superior chemical and photothermal stabilities, filming properties, and, more importantly, its hydrophilic -SO_3_^–^ ionic-cluster regions, hydrophobic fluorocarbon regions, and the interfacial regions that are formed between them that makes it more suitable than other polymers for sensing applications [30]. It has been reported that metalloporphyrins occupy the hydrophobic and interfacial regions of the Nf film due to their hydrophobic nature [31]. Moreover, the polyvalent ampholytic nature of the tetrakis(4-nitrophenyl)porphyrin (TNPP) inner core provides sensitivity toward bases and acids [32,33]. Thus, the advantages of the novel Nf-TNPP thin film include its high activity, low cost, and high selectivity for EDA and H_2_S gases.

While toxic gases can be examined using conventional analytical methods, such as GC/MS and AAS, leading to the highly sensitive and well resolved detection and quantification of analytes [34,35], these methods suffer from some disadvantages; they are generally costly, require experienced operators, and are inconvenient for real-time monitoring. Since the 1980s, researchers in the field of optical communications have paid close attention to the study and application of optical-waveguide (OWG) sensors [36]. OWG sensors have been developed for the measurement of trace amounts of a variety of gases [37,38,39,40]. To the best of our knowledge, EDA and H_2_S gases have, to date, not been determined utilizing OWG methods, even though OWG sensors exhibit several desirable features, including portability, potential for high sensitivity, anti-electromagnetic interference properties (they suffer little or no interference in the waveguide element of the sensor), fast response-recovery times, and intrinsic safety for detection at room temperature when compared to other sensors. In this work, TNPP and Nf-TNPP were used as sensing elements embedded in an OWG sensor to determine the presence of analytes through changes in absorption properties. A highly sensitive and rapidly responding/recovering Nf-TNPP sensor was subsequently fabricated and applied to the detection of low concentrations of EDA and H_2_S gases. Finally, porphyrin protonation and deprotonation in Nf-TNPP, and the mechanism that is responsible for the diversity of these structures, are discussed.

## 2. Methods

### 2.1. Synthesis and Characterization of Meso-tetrakis(4-Nitrophenyl) Porphyrin (TNPP)

TNPP was synthesized using a procedure modified from that reported by Fasalu et al [41] Pyrrole (0.5 mL, 7.2 M) and 4-nitrobenzaldehyde (1.09 g, 7.2 M) were taken in a 1 L three-necked round-bottom flask and dissolved in 500 mL CHCl_3_. The solution was purged with nitrogen gas for 10 min. Then, the acid catalyst BF_3_-Et_2_O (0.31 mL, 2.5 M) was dispensed into the mixture. After 2 h of stirring, the reaction was quenched by the addition of *N,N*-diethylethanamine (0.35 mL, 2.5 M), and then, 2,3-dichloro-5,6-dicyano-1,4-benzoquinone (DDQ, 1.63 g, 7.2 M) was added. The reaction mixture was further mixed for another hour at 65 °C. The solvent was evaporated by using a rotary evaporator, and then purified by column chromatography using neutral alumina and chloroform/methanol (95:5) as the eluent. The yield of TNPP was 219 mg (16%). ^1^H NMR (Bruker AVANCE DMX400, 400 MHz, CDCl_3_, Billerica, MA, USA) δ = 8.58–8.01 (m, 4H), 7.71 (d, J = 34.0 Hz, 2H), 6.90–6.12 (m, 2H), 5.19 (s, 4H), 1.25 (dd, J = 20.6, 13.3 Hz, 12H), 0.85 (s, 2H).

### 2.2. Preparation of Nafion Solution

Nafion NR50 mesh beads were sonicated for 3 h in an ethanol solution, and then placed in a vacuum drying oven for 12 h at 60 °C in order to further purify the beads from residues of small polymers and fragments that were left over from manufacture. To a 25 mL high-pressure reaction vessel, 0.1 g of Nafion was added after 5 mL ethanol and 5 mL distilled water were completely mixed. This was then taken into the vacuum drying oven again for 4 h at 230 °C. The reaction vessel was removed from the oven and cooled to room temperature. The result was a clear and colourless solution, containing 0.01 wt % of Nafion.

### 2.3. Preparation and Analysis of Sensing Element

**Scheme 1:**
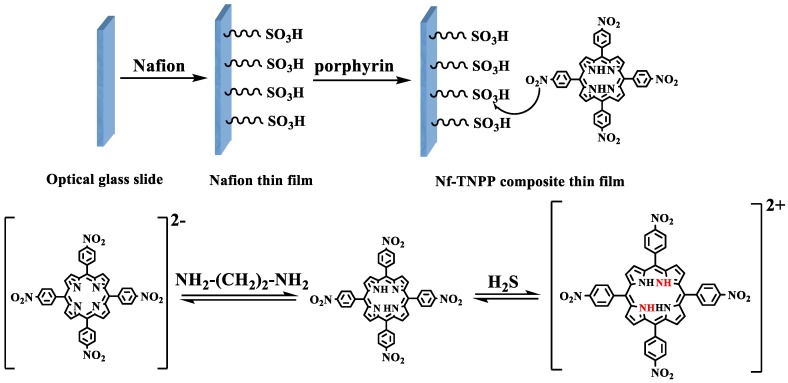
Stepwise fabrication of the functionalized Nf-TNPP-composite thin film and possible interactions with analytes.

Before fabrication of the sensing element, a potassium ion exchanged glass slide was washed with acetone and ethanol. After the drying process, the glass slide was coated with the previously prepared Nafion solution (0.01 wt %) at 2200 rpm for 30 s using a spin coater, followed by desiccation for 1 h at 70 °C. Finally, a certain concentration of the TNPP solution (0.66 mg cm^−3^) was immobilized on the Nafion film OWG surface by spin coating at 2000 rpm for 30 s (Scheme 1), and was then used for gas detection.

A Keysight 5500 Atomic force microscopy (AFM, Bruker, Billerica, MA, USA) was used to characterize the surface morphology and thickness of both TNPP and functionalized Nf-TNPP thin films. The acquired images were collected in the tapping mode with lateral dimensions ranging from submicrometre range to several micrometres.

Further, the Nf-TNPP film was characterized by UV-Vis and emission (FTIR-650, Beijing Kaifeng Fengyuan Technology Co., Ltd., Beijing, China) spectroscopy before and after exposure to the analyte gases. The absorbance measurements were conducted using a UV-1780 spectrophotometer (Shimadzu Technology Co., Ltd., Beijing, China) on solutions deposited on an optical glass substrate.

## 3. Sensing Apparatus and Procedure

Gas sensing experiments were performed using a homemade OWG detection system, in which the sensor was mounted with prism-coupler feedthroughs. This gas sensor system is similar to that described in previous reports [42,43,44]. The gas-testing apparatus [45] was composed of a compressed air source and an Nf-TNPP-film OWG gas-sensitive element, a laser source, a flow meter, a diffusion tube, a light detector, and a recorder. A gas-mixing manifold, into which VOCs and a stream of pure air were introduced, was used for mixing and introducing the mixture into the flow cell, which enclosed the waveguide sensor. The Nf-TNPP thin-film-sensor device inside the flow cell (2 × 1 × 1 cm) was mounted on a rotational stage possessing X-Y-Z translation capabilities. Two different semiconductor laser beams (532 nm for EDA, 650 nm for H_2_S) were introduced into the Nf-TNPP thin-film-based OWG using a prism coupler; these beams exited through another prism coupler. The intensity of the output light was monitored using a photomultiplier detector, and the signal was recorded by a computer. For each measurement, a fresh syringe was used to inject 20 cm^3^ of the gas sample into a new flow cell, after which it was vented. All of the experiments were performed at room temperature during which pure N_2_ (99.99%) was introduced into the cell at a constant flow rate of 50 cm^3^ min^–1^ in order to transfer the sample gas to the sensor.

Different concentrations of analytes were prepared in two steps. First, standard H_2_S gas was obtained by reacting a given amount of FeS with HCl at room temperature at atmospheric pressure. The gas that was produced in this manner was allowed to flow into a sealed standard vessel (600 cm^3^). Standard EDA gas was produced by vaporizing a given amount of EDA solution (99.5%) inside a sealed standard vessel (600 cm^3^). The concentrations of the H_2_S and EDA gases, calibrated using a commercial H_2_S gas detection tube (working range: 2–200 ppm, Gastec, Beijing Municipal Institute, Beijing, China) and an EDA gas detection tube (Gastec, GV-100, Kobe, Japan), were approximately equal to the calculated values. Second, different amounts of the standard H_2_S and EDA gases were diluted with pure air in a second standard vessel (600 cm^3^) to obtain the desired concentrations. Using this standard-vessel dilution method, very low concentrations of H_2_S and EDA (in the ppb range) could be produced.

## 4. Result and Discussion

**Figure 1:**
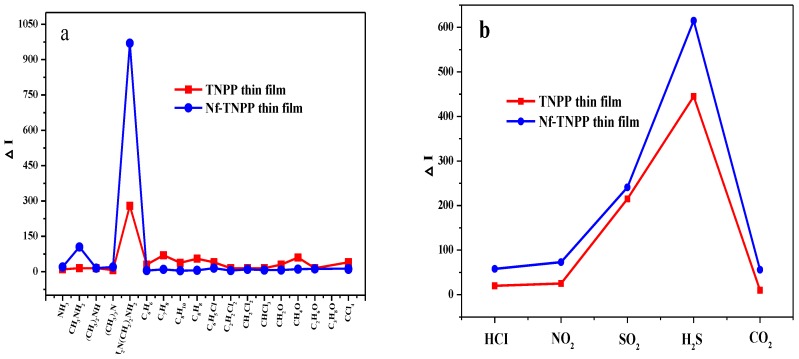
Responses of tetrakis(4-nitrophenyl)porphyrin (TNPP) and Nafion membrane (Nf)-TNPP thin film/K^+^-exchanged OWG sesnors upon exposure to (**a**) VOCs and (**b**) inorganic gases at room temperature (gas concentrations: 100 ppm).

Previous studies investigated the interactions of porphyrins bearing free bases with analytes and quantitatively determined acidic and alkaline gases on the basis of UV-vis spectral shifts [46]. In the present study, we determined the concentrations of EDA and H_2_S gases from Soret-band absorbances using the OWG technique. The gas-sensing responses of Nf-TNPP and TNPP films to 100 ppm of VOCs and inorganic gases are shown in Figure 1a,b, respectively. The response factor of the sensor is defined by: Δ*I* = *I_gas_* − *I_air_*, where *I_gas_* and *I_air_* are the signal intensities of the gas and ambient air, respectively. Figure 1 reveals that the Nf-TNPP-film-based OWG sensor is more sensitive and more stable toward EDA and H_2_S than the TNPP-film-based sensor. This is ascribable to electrostatic interactions between the four -NO_2_ functional groups on the porphyrin and the -SO_3_^–^ groups of the Nafion film [46]. Consequently, the Nf-TNPP film was further investigated.

Selectivity is a contributing factor to gas-sensing performance, since good selectivity determines whether or not target analytes are detectable in a multi-component gas environment [47]. Here, the sensor responses of the Nf-TNPP film to 100 ppm VOCs and inorganic gases, including HCl, H_2_S, SO_2_, NO_2_, and CO_2_, were measured at room temperature. Clearly, as shown in Figure 1, Nf-TNPP exhibited a higher response to EDA (at 532 nm) and H_2_S (at 650 nm) among similar analytes. The typical response of this sensor to EDA was approximately six-times greater than its response to methylamine, and its response to H_2_S was about 2.7-times greater than its response to SO_2_. These results reveal that the Nf-TNPP film exhibits good selectivity to EDA and H_2_S gases.

**Figure 2:**
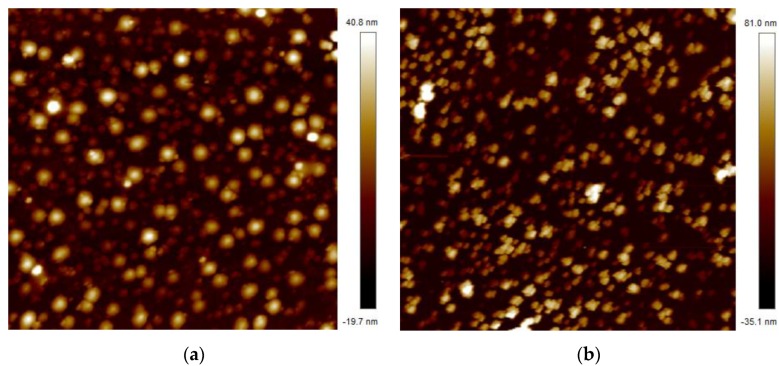
Atomic force microscopy (AFM) images of the spin-coated (**a**) Nf-TNPP thin-film surface, and (**b**) TNPP thin-film surface (area: 10 × 10 μm^2^).

AFM, performed using a Multimode 8 system, was used to characterize the surface morphologies of the TNPP and Nf-TNPP thin films (Figure 2). The surface of the Nf-TNPP thin film exhibits higher molecular ordering when compared to that of the TNPP thin film; the average surface roughnesses of the Nf-TNPP and TNPP sensing films were determined to be 9.97 nm and 20.4 nm, respectively. In addition, the peaks of the Nf-TNPP film are less high and the valleys less deep (i.e., the film is flatter, Figure 2a) than those of the TNPP film (Figure 2b), owing to the presence of functional groups on the film surface [48,49,50,51]. The strong interactions between the Nf and TNPP are recognizable by a noticeable conformational change in the morphology of the film layer, which highlights the inclusion of TNPP molecules into the Nf structure; this also indicates that the TNPP molecules are well distributed across the surface of the Nf film. Functionalization of the Nf surface with TNPP molecules significantly affects the OWG output-light intensity (Figure 1).

**Figure 3:**
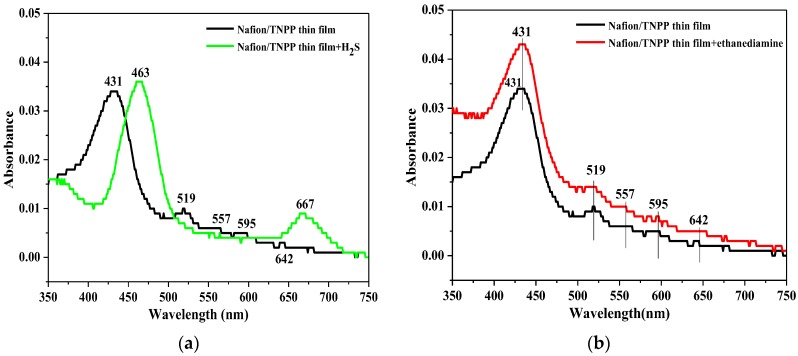
Absorption spectra of the Nf-TNPP film before and after exposure to (**a**) hydrogen sulfide (H_2_S) and (**b**) Ethanediamine (EDA) gas vapor.

After exposure to H_2_S, the Nf-TNPP film, which was initially black in color, turned green, whereas its color changed to red upon exposure to EDA gas; the associated UV-vis spectral changes are shown in Figure 3. When the Nf-TNPP film was exposed to H_2_S gas, remarkable changes were observed in the visible region of its absorption spectrum. The Soret (B) band of the Nf-TNPP film was observed to (red) shift from 431 nm to 463 nm, while the four Q bands, at 519, 557, 595, and 642 nm, merged into a single Q band at 667 nm (Figure 3a). No noticeable shifts in the positions of the B band at 436 nm and the Q-bands at 480–750 nm were observed when the film was exposed to EDA gas; however, their absorption intensities strengthened noticeably (Figure 3b). These changes are attributable to the protonation of TNPP by H_2_S gas. On the other hand, when the same film is exposed to EDA gas, the porphyrin becomes deprotonated (see Scheme 1 for possible interactions between the film and the analytes) and coordinated to the residual –SO_3_^–^ groups to enhance the intensities of the absorption peaks of the film. The absorption changes that are observed for the Nf-TNPP film upon exposure to analytes also reveal that readily available semiconductor laser beams should be effective light sources for the Nf-TNPP film OWG sensor. The refractive index and thickness of the OWG device, as measured by an SGC-10 ellipsometer, were 1.68 and 138 nm, respectively. A theoretical calculation [52] reveals that a 138 nm thick Nf-TNPP film should support the TE_0_ mode of the optical waveguide [53]. Propagation of the TE_0_ mode in the Nf-TNPP-film-based OWG sensor by analyte gases was examined by laser-beam prism coupling.

Optical-waveguide gas sensors rely on two principles. Firstly, the absorbance changes exhibited by the film are directly related to interactions with the detected gas, and, secondly, the sensor responds to changes in the absorbance of the film that are a consequence of interactions with analytes. In order to elucidate the response of the sensor to H_2_S and EDA, and the suitabilities of the chosen laser-beam wavelengths for OWG detection, the response of the sensor was investigated at 532 and 650 nm using the same concentrations of VOCs and inorganic gases (Figures S1–S4). The absorbance changes and responses of the sensor to H_2_S and EDA at 532 and 650 nm are listed in Table 1, which reveals that larger absorbance changes are observed in response to each analyte at its preferred wavelength. At 532 nm, the response of the sensor to EDA is 9.7 times greater than it is to H_2_S, while at 650 nm, the response of the sensor to H_2_S is about 2.5 times greater than to EDA. Therefore, these two semiconductor laser wavelengths were used to detect the target analytes: 532 nm for EDA and 650 nm for H_2_S.

**Figure 4:**
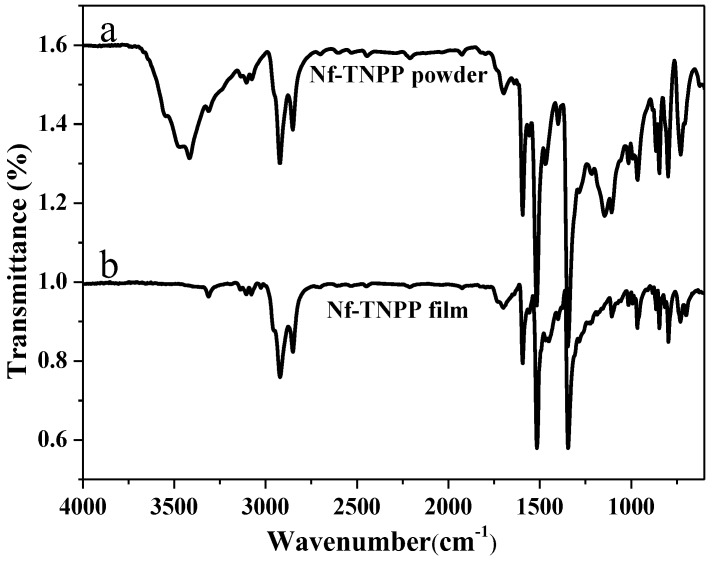
FT-IR spectra of TNPP: (a) powder and (b) thin film.

Figure 4 displays FT-IR spectra of the TNPP powder (see in Figure 4a) and thin film (see in Figure 4b). In the powder, the ν_N-H_ stretching band of TNPP appears at 3416 cm^–1^, while CH_2_ asymmetric and symmetric stretching bands of the alkyl chain appear at 2922 cm^–1^ and at 2851 cm^–1^, respectively. The spectrum of the powder also exhibits a middle strong band at 1594 cm^–1^, which can be attributed to the ν_C = C_ vibration band of pyrrole, and two strong bands at 1145 and 1107 cm^–1^ that can be attributed to the ν_C = C_ and ν_C = N_ of the porphyrin macrocycle, respectively. Moreover, the spectrum of the powder also shows two weaker bands at 843 and 798 cm^–1^, which can be attributed to ν_C-C_ of the two substitutes of the phenyl benzene ring. When compared with the peaks obtained from the powder, the absorption peaks of the TNPP thin film are very similar in the region between 4000 and 600 cm^–1^. However, the absorption bands of ν_N-H_ at 3416 cm^–1^ and in the macrocycle at 1145–1107 cm^–1^ ν_C = C_ (or ν _C= N_) are visibly different in the spin-coated film. This indicates that the molecules in the film are arrayed more closely [54].

**Figure 5:**
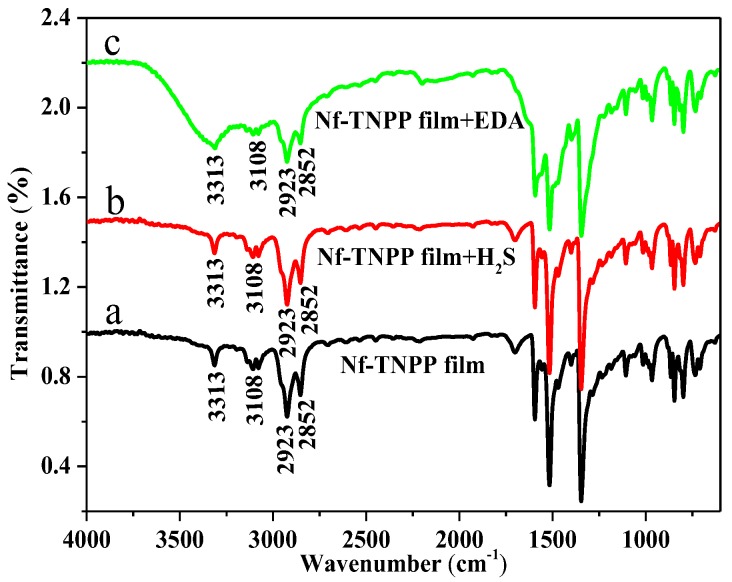
FT-IR spectra of the Nf-TNPP thin film (a) before, and after exposure to (b) H_2_S and (c) EDA vapor.

Generally, the sensing mechanism that is used by gas sensors involves the absorption/desorption of analytes at their surfaces. Depending on the chemical nature of the sensing materials and the analytes, absorption/binding can occur through weak and/or strong chemical interactions, such as van der Waals, dipole-dipole, hydrogen bonding, and π-π interactions [21]. In this paper, in order to examine possible interactions between the composite thin film and detected gases, the gas response of the thin film to saturated H_2_S or EDA vapor was examined by FT-IR (Fourier Transform Infrared Spectoscopy) spectroscopy, the results of which are displayed in Figure 5. Upon exposure to each analyte, strong hydrogen bonds may form between the –NH– or –N– groups of the pyrrole ring and the hydrogen atoms of H_2_S or the –NH_2_ groups of EDA, which may result in proton transfer. The spectra of the thin film before (Figure 5a) and after exposure to H_2_S gas (Figure 5b), reveal that the peak positions and the absorption intensities are essentially the same, indicating that the functional groups of the film are effectively unchanged as a result of their interactions with H_2_S gas. However, upon exposure to EDA vapor, a relatively strong and broad stretching band appeared at 3313 cm^–1^ (Figure 5c), which is partly due to primary amines that are formed through interactions between the –NH– group of the pyrrole ring and the secondary amines of the EDA as a result of the acidity of TNPP, and partly due to the ν_N-H_ stretching band of the EDA vapor.

**Figure 6:**
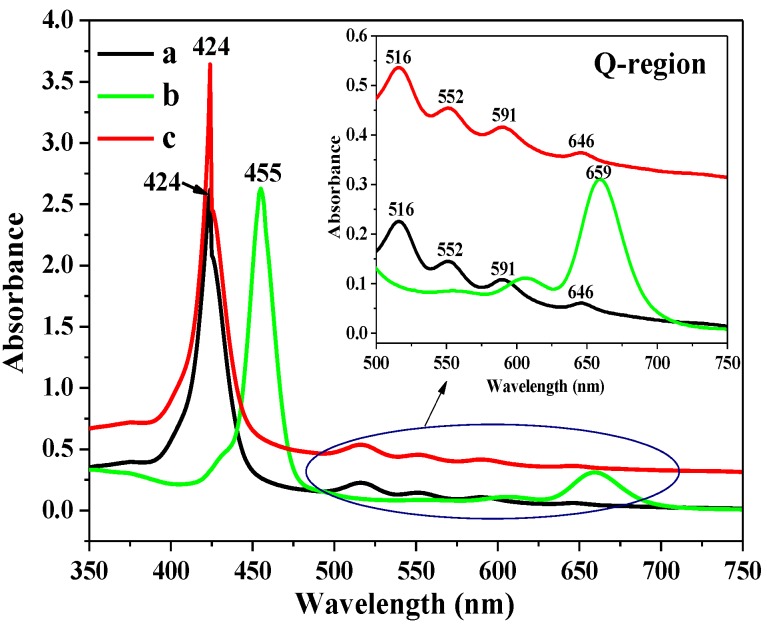
UV-vis absorption spectra of TNPP solutions (a) before and after exposure to (b) H_2_S and (c) EDA vapors.

The TNPP molecule is square in shape, with two imine-type nitrogen atoms in the center of the ring. Protonation occurs at these two nitrogen atoms under certain conditions. In order to elucidate this phenomenon, sample solutions of TNPP were characterized by UV-vis spectroscopy in chloroform, before and after exposure to H_2_S and EDA gases, the results of which are displayed in Figure 6. The TNPP solution exhibits a B band at 424 nm and Q bands from 500 to 650 nm in its UV-vis spectrum. These bands are attributed to transitions between the highest and lowest molecular orbitals, and correspond to π → π transitions. The intensity of the B-band absorption peak of TNPP increases upon exposure to EDA gas, leading to an increase in molar absorptivity (Figure 6c). On the other hand, the B band becomes red shifted, from 424 to 455 nm (Figure 6b), when the solution is exposed to H_2_S, and the four Q bands strengthen and merge into a single band. These spectral changes are observed both in solution, and in the solid film, and are in agreement with reports in the literature [55]. The red shift of the Soret band and the enhancement of the Q band at 659 nm indicate that the inner nitrogen atoms of the TNPP ring become protonated by H_2_S, leading to enhanced porphyrin conjugation and an overall reduction in the energy of the porphyrin system.

**Figure 7:**
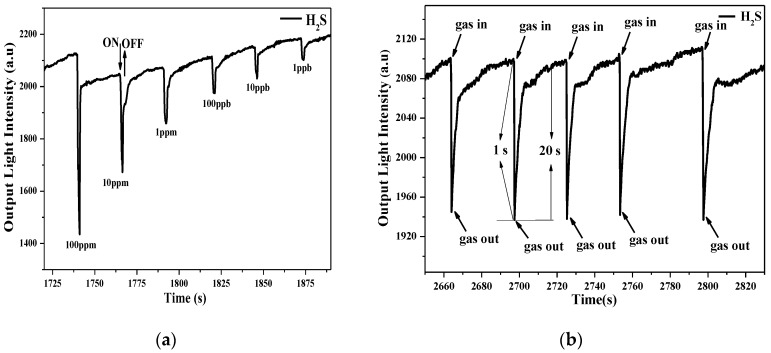
Nf-TNPP-film-based/K^+^-rxchanges optical-waveguide (OWG)-sensor performance. (**a**) Response reproducibility and (**b**) response-recovery curve over five cycles for 100 ppb H_2_S gas.

**Figure 8:**
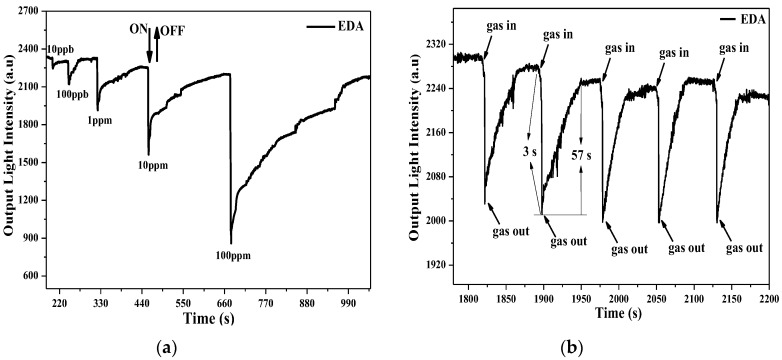
Nf-TNPP-film-based/K^+^-rxchanges OWG-sensor performance. (**a**) Response reproducibility and (**b**) response-recovery curve over 5 cycles for 100 ppb EDA gas.

For an OWG sensor, any variation in the optical properties of the thin film alters the intensity of absorbed light, and, consequently, the amount of transmitted light. To explore sensor reversibility, we prepared a sensor using a film that was composed of the dipotassium salt of Nf-TNPP, in which both acidic protons were exchanged for K^+^; typical responses of this sensor to H_2_S and EDA vapor are shown in Figure 7. As can be seen in Figure 7, when continuous air flows during the entire detection process, the intensity of the transmitted light decreases in response to the presence of H_2_S and EDA gases in the 10 ppb to 100 ppm range. The as-prepared OWG device exhibited an excellent response (ΔI = 77) to 1 ppb H_2_S, with a signal-to-noise ratio (SNR) of 7.8 dB, and an excellent response (ΔI = 79) to 10 ppb EDA, with an SNR of 31 dB. In addition, the response/recovery times of each gas were calculated. These times are two fundamental and important factors that, together with response, are considered to define gas-sensing performance. The response time is defined as the time required to establish 90% of the equilibrium value following exposure to the analyte, and recovery time is the time required for the sensor to return to within 10% of the initial response in air upon release of the analyte gas [56]. Figure 7 and Figure 8 reveal that, at commencement, the gas sensor exhibits a constant and stable baseline. When each analyte gas was introduced into the flow cell, the intensity of the corresponding transmitted light decreased. However, when the flow cell was refilled with pure N_2_, the signal returned to the original baseline value. The response and recovery times were 1 s and 20 s, and 3 s and 57 s, to 100 ppb H_2_S (Figure 7b) and EDA (Figure 8b), respectively. Good Nf-TNPP-film OWG sensor reproducibilities were demonstrated by successively exposing it to 100 ppb H_2_S or EDA gases over five cycles at room temperature (Figure 7b and Figure 8b). In addition, the response-recovery times of EDA were about three times longer than those of H_2_S at the same concentrations, which may due to the viscosity of EDA gas [57]. At this concentration, the relative standard deviations of the transmitted light intensity are ±4.29 for H_2_S and ±5.10 for EDA.

When the sensor was exposed to analyte gases, the optical properties of the sensitive element were changed. As shown in the following equation, the intensity of transmitted light is related to the absorbance coefficient, thickness, and refractive index of the sensitive thin layer:

(1) $$I=I_{0}\left(1-a{Nd}_{e}\right)$$

In which *I* denotes the intensity of the output light, *I*_0_ is the intensity of input light, *a* is the absorbance coefficient of the sensitive thin layer, *N* is the refractive numbers of the guided light on the surface of the OWG with length *L*, and *d_e_* is the real propagated distance of light, which is related to the thickness of the sensitive thin layer. Based on the above formula, it is apparent that when the thickness or the absorbance coefficient of the sensitive thin layer decreases, the intensity of the output light will increase.

**Figure 9:**
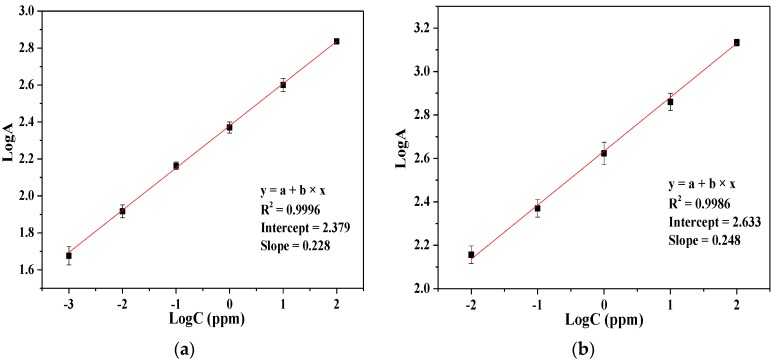
Relationship between the response intensity and the concentration of (**a**) H_2_S and (**b**) EDA vapour.

The response intensity of the sensor, defined LogA = Log(*I*_0_ − *I*_t_), where *I*_0_ is the initial output light intensity and *I_t_* is the lowest point of the output light intensity, corresponding to before and after the injection of analytes into the flow cell, respectively. The calibration curve of the Nf-TNPP film OWG sensor was obtained by plotting the response intensity *LogA* against the concentration of the analyte gases, and the linear relationships between them are given in Figure 9a, and the relationships between increasing concentrations of H_2_S (see in Figure 9a) and EDA (see in Figure 9b) gases and signals were found to be linear. We therefore acquire the following relations: LogA_hydrogen sulfide_ = (2.38 ± 0.004) + (0.23 ± 0.002) C(H_2_S), R = 0.99, and LogA_EDA_ = (2.63 ± 0.008) + (0.25 ± 0.004) C(EDA), R = 0.99.

## 5. Conclusions

In summary, TNPP was synthesized and dissolved in a solution of Nafion, after which it was used to prepare Nf-TNPP thin films by spin coating. Absorption and ^1^H NMR spectra of TNPP were acquired. The surface morphologies of the TNPP and Nf-TNPP thin films were investigated by AFM, which revealed that the surface of the Nf-TNPP film was smoother than that of TNPP. Planar OWG sensors of K^+^-exchanged TNPP and Nf-TNPP thin films on glass were prepared and were used to detect VOCs. Among them, the Nf-TNPP thin film exhibited preferable optical responses to H_2_S and EDA at room temperature. The sensor also exhibited noticeable responses to EDA and H_2_S in the presence of amines and some inorganic gases (Figure S5). We developed a sensitive, fast, and simple optical sensor for the determination of H_2_S and EDA based on the absorption spectrum of Nf-TNPP. In addition, possible interactions between the sensor and the analytes are discussed in conjunction with FT-IR and UV-vis spectroscopy. The sensor film exhibited high sensitivity and selectivity toward H_2_S and EDA gases, and detected ppb concentration levels of these gases through the use of two different semiconductor laser-beam wavelengths.

## Figures and Tables

**Table 1:** Absorbance changes (ΔAbs = |Abs_gas_ − Abs_film_|) and responses (ΔI) of the Nf-TNPP sensor upon exposure to H_2_S and EDA at different laser-beam wavelengths.

Gas Sample	ΔAbs (532 nm)	ΔAbs (650 nm)	ΔI (532 nm)	ΔI (650 nm)
H_2_S	0.001	0.005	100	530
EDA	0.005	0.002	970	212

## Supplementary Materials

The Supplementary Materials are available online at http://www.mdpi.com/1424-8220/17/12/2717/s1.
