# Correction: Gulimire Tuerdi, Nuerguli Kari, Yin Yan, Patima Nizamidin and Abliz Yimit *. A Functionalized Tetrakis(4-Nitrophenyl)Porphyrin Film Optical Waveguide Sensor for Detection of H_2_S and Ethanediamine Gases. *Sensors* 2017, *17*, 2717

**DOI:** 10.3390/s20051522

**Published:** 2020-03-10

**Authors:** Gulimire Tuerdi, Nuerguli Kari, Yin Yan, Patima Nizamidin, Abliz Yimit

**Affiliations:** College of Chemistry and Chemical Engineering, Xinjiang University, Urumqi 830046, China; gulmirat@126.com (G.T.); nurri7695@163.com (N.K.); yanyinwx@163.com (Y.Y.); patima207@aliyun.com (P.N.)

The authors wish to make the following corrections to this paper [1]:

1. The authors are sorry to report that some of the ^1^H NMR data reported in their recently published paper [1] were incorrect. Our understanding at that time was to determine the total number of hydrogen atoms of the compound according to the ratio of the number of spectral areas. Consequently, the authors wish to at this time make the following corrections to the paper:

^1^H NMR ((JEOL) JNM-ECA600, 600 MHz, Chloroform-d) δ 8.82 (s, 8H), 8.67(d, J = 7.8 Hz, 8H), 8.39 (d, J = 7.8 Hz, 8H), −2.83 (s, 2H) (Supplementary Materials Figure S1); this result is in good agreement with previous reports [42,43]. 

In order to further characterize the purity of the material, we carried out an elemental analysis measurement. Firstly, three samples were weighed for testing, as shown in Supplementary Materials Figure S2. It can be seen that according to the percentage mass concentration of C, N and H, the averages were 65.33%, 11.11% and 4.64%, respectively. Then, from the molecular formula C_44_H_26_N_8_O_8_ of TNPP (M = 794 g/mol) the atom mass ratio obtained was C:H:N:O = 66.43%:3.27%:14.09%:16.10%. Finally, based on the comparison of the results of elemental analysis detection, if H_2_O and CD_3_Cl were impurities, then the purity of synthesized TNPP determined by N element was, therefore, the following: purity = (11.11%/14.09%) = 78.85%.

2. There are two mistakes in this article [1]. The drying process, “The composite film was heat-treated at 80 °C for 10 min to allow desorption of solvents trapped on the film surface, and then it was dried in a vacuum oven for 24 h at room temperature” was missing on page 3, lines 27 and 28. The model of the instrument “Keysight 5500 atomic force microscopy (AFM, Bruker, Billerica, MA, USA)” should be “JNM-ECA600 spectrometer (JEOL, Japan)” on page 3, line 29. 

3. We have found the following inadvertent error on page 11, lines 7 and 8: “The sensor also exhibited noticeable responses to EDA and H_2_S in the presence of amines and some inorganic gases (Figure S5)”. It should be, “The most serious weakness of current sensor technology is sensitivity to changes in humidity. Muthukumar et al. examined the sensitivity of Nf-TNPP film to ~20%, ~50% and ~80% relative humidity in the presence of NH_3_ gas. The film showed strong stability and sensitivity to the same concentration of NH_3_ gas, which means the as-developed Nf-TNPP gas sensor showed only a small influence of humidity [29]. In general, ambient humidity effected small changes to the sensitivity and selectivity of the gas sensor developed in the current study. The sensor also exhibited noticeable responses to EDA and H_2_S in the presence of amines and some inorganic gases. The obtained responses showed that the reversibility of the sensing film was affected by interfered gases (Supplementary Materials Figure S7). The complete recovery of the sensing film could not be achieved and the baseline also varied“ (page 11, lines 5–14 in our paper published in *Sensors* [1]).

4. On page 7, lines 5 and 6, “The response of the sensor was investigated at 532 and 650 nm using the same concentrations of VOCs and inorganic gases (Figures S1–S4)” has been changed to “the response of the sensor was investigated at 532 and 650 nm using the same concentrations of VOCs and inorganic gases (Supplementary Materials Figures S3–S6)”. 

5. Due to the addition of new literature, the order of the original citations has changed, namely, “[42–44]” has been changed to “[44–46]” on page 3, line 46; “[45]” has been changed to “[47]” on page 3, line 46; “[46]” has been changed to “[48]” on page 4, line 25 and line 33; “[47]” has been changed to “[49]” on page 4, line 36; “[48–51]” has been changed to “[50–53]” on page 5, line 7; “[52]” has been changed to “[54]” on page 6, line 12; “[53]” has been changed to “[55]” on page 6, line 13; “[54]” has been changed to “[56]” on page 7, line 24; “[55]” has been changed to “[57]” on page 8, line 26; “[56]” has been changed to “[58]” on page 9, line 14; and [57] has been changed to [59] on page 9, line 23. All these changes were marked in red in the main body, and citations were corrected in the “References” section of the corrected paper.

42. Kangwanwong, T.; Pluempanupat, W.; Parasuk, W.; Keenan, H.E.; Songsasen, A. Using 5,10,15,20-tetrakis(4-nitrophenyl)porphyrin as a fluorescent chemosensor to determine R33u^3+^. *Sci. Asia* **2012**, *38*, 278–282.
43. Meng, G.G.; James, B.R.; Skov, K.A., Porphyrin chemistry pertaining to the design of anti-cancer drugs; part 1, the synthesis of porphyrins containing meso-pyridyl and meso-substituted phenyl functional groups. *Cana. J. Chem.* **1994**, *72*, 2447–2457.

These changes have no material impact on the conclusions of our paper. We apologize to our readers.
