# Supplementary material for: A Functionalized Tetrakis(4-Nitrophenyl)Porphyrin Film Optical Waveguide Sensor for Detection of H2S and Ethanediamine Gases
Source: Sensors (Basel). 2017 Nov 24;17(12):2717. doi: 10.3390/s17122717 (PMC5751706; doi:10.3390/s17122717)
Supplement: Supplementary file 1 [file sensors-17-02717-s001.pdf]

Supplementary Materials

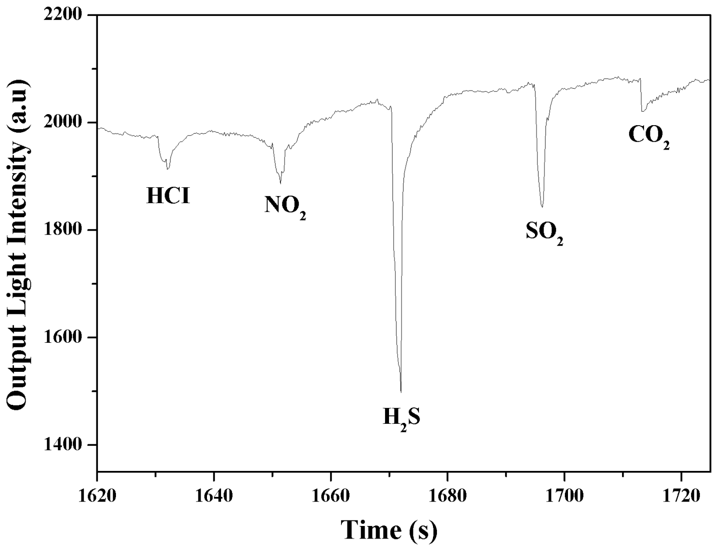

Figure S1. The Nf-TNPP film OWG sensor response to 100 ppm of inorganic gases at 650 nn

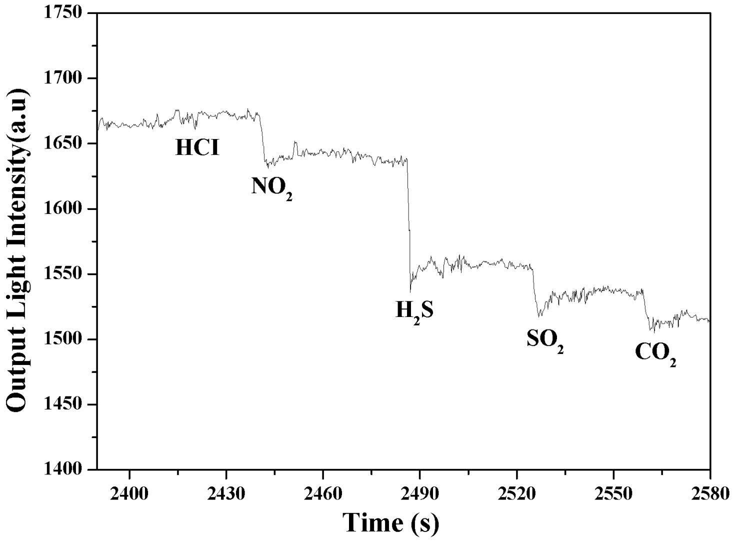

Figure S1. The Nf-TNPP film OWG sensor response to 100 ppm of inorganic gases at 532 nn

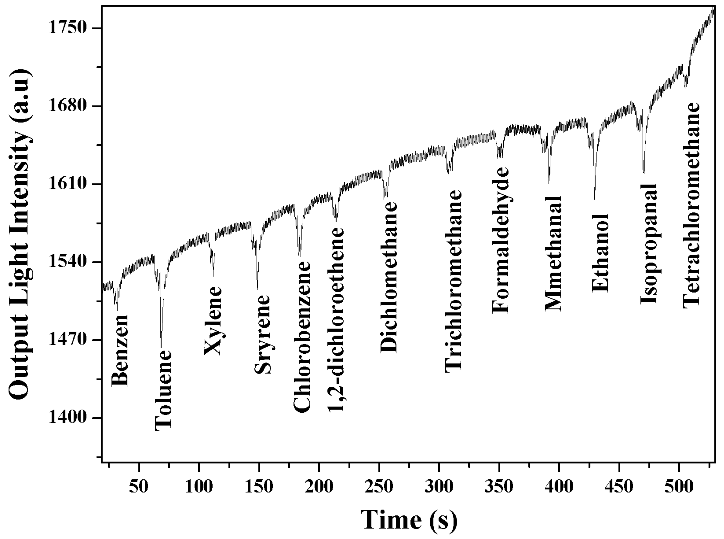

Figure S2. The Nf-TNPP film OWG sensor response to 100 ppm of VOCs at 650 nn

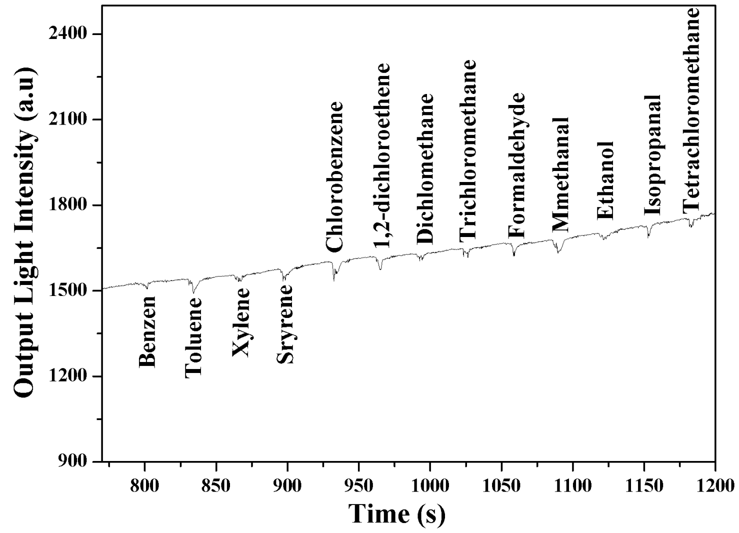

Figure S2. The Nf-TNPP film OWG sensor response to 100 ppm of VOCs at 650 nn

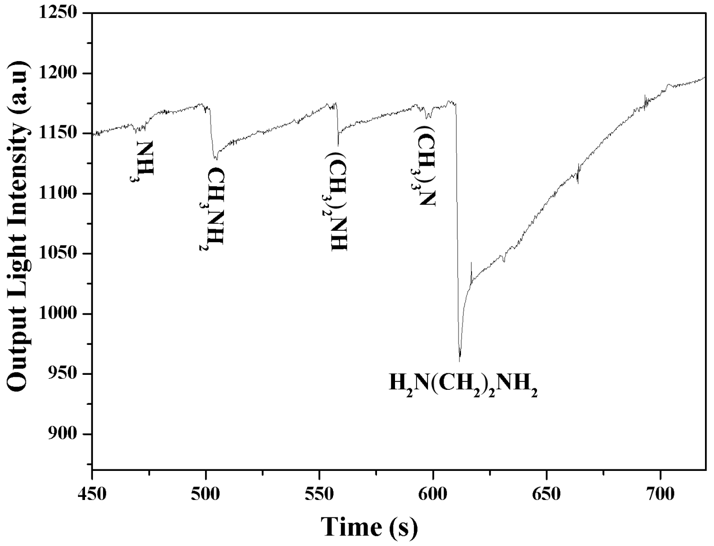

Figure S3. The Nf-TNPP film OWG sensor response to 100 ppm of amines at 650 nn

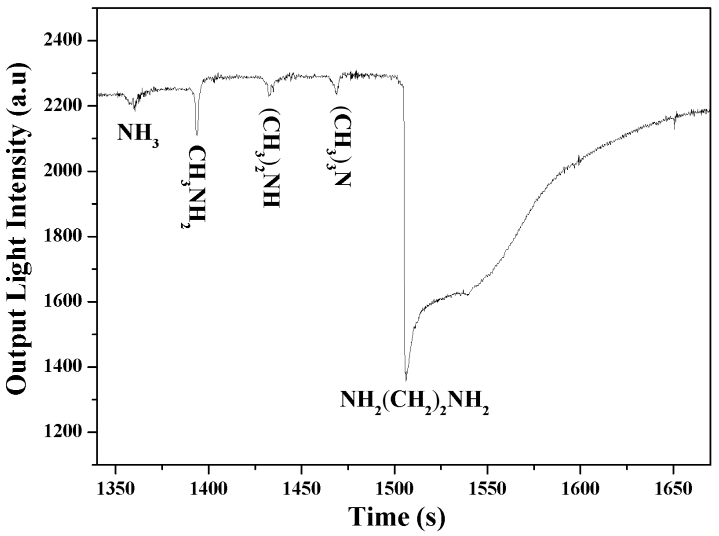

Figure S3. The Nf-TNPP film OWG sensor response to 100 ppm of amines at 532 nn

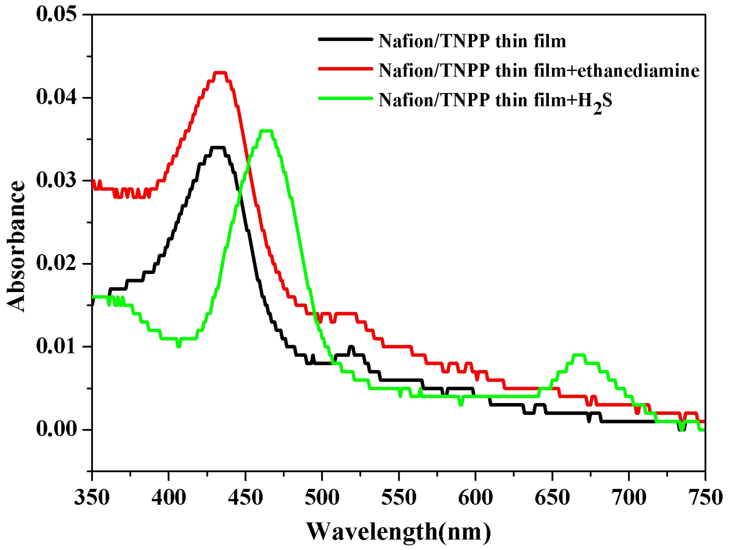

Figure S4. Absorption spectra of Nf-TNPP film before and after exposure to H_2S and EDA gas vapours (350-750 nm)

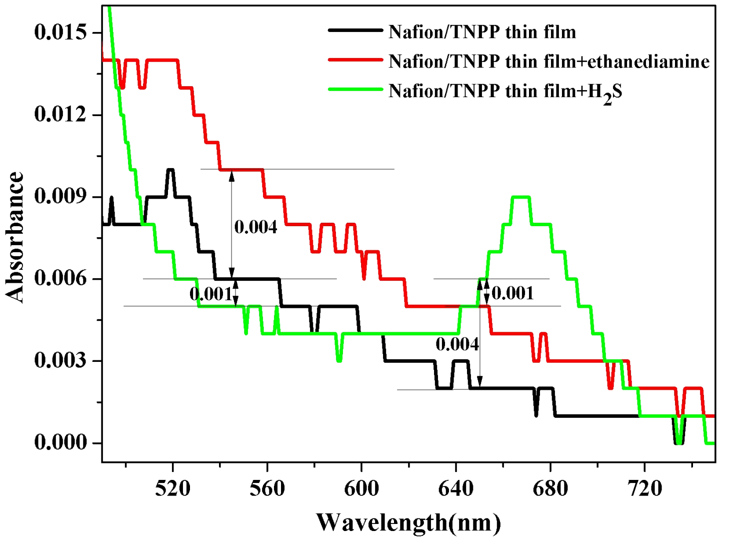

Figure S4. Absorption spectra of Nf-TNPP film before and after exposure to H_2S and EDA gas vapours (490-750 nm)

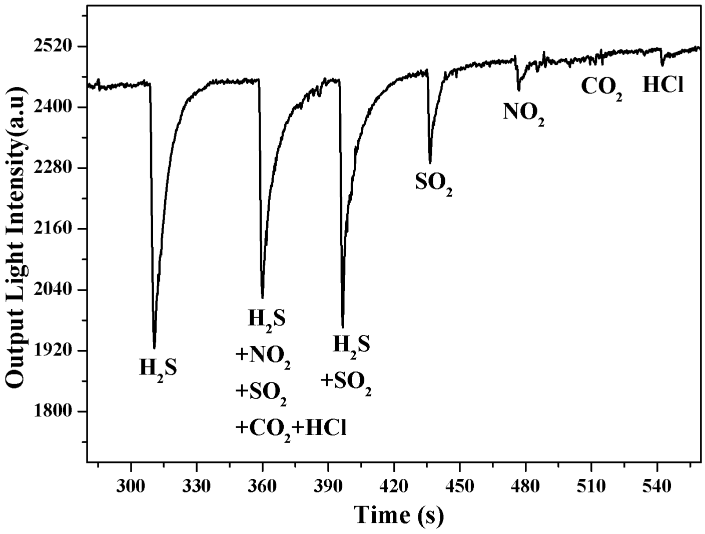

Figure S5. The Nf-TNPP film OWG sensor response to 100 ppm of H2S in presence of interfereing gases at 650 nn

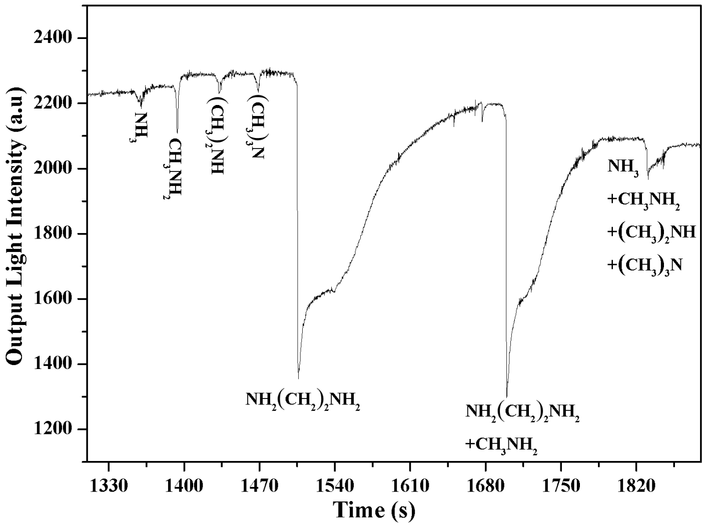

Figure S5. The Nf-TNPP film OWG sensor response to 100 ppm of EDA in presence of interfereing gases at 532 nn
